# Hesperidin, a Potential Antiviral Agent against SARS-CoV-2: The Influence of Citrus Consumption on COVID-19 Incidence and Severity in China

**DOI:** 10.3390/medicina60060892

**Published:** 2024-05-28

**Authors:** Adam Kowalczyk

**Affiliations:** Department of Pharmacognosy and Herbal Medicines, Faculty of Pharmacy, Wroclaw Medical University, 50-556 Wrocław, Poland; adam.kowalczyk@umw.edu.pl

**Keywords:** hesperidin, SARS-CoV-2, COVID-19, antiviral agents

## Abstract

This review examines hesperidin, a citrus bioflavonoid, as a potential antiviral agent against SARS-CoV-2. The COVID-19 pandemic has demanded an urgent need to search for effective antiviral compounds, including those of natural origin, such as hesperidin. The review provides a comprehensive analysis of the chemical properties, bioavailability and antiviral mechanisms of hesperidin, particularly its potential efficacy against SARS-CoV-2. A review of databases, including PubMedPico, Scopus and Web of Science, was conducted using specific keywords and search criteria in accordance with PRISMA (Re-porting Items for Systematic Reviews and Meta-Analysis) guidelines between 2020 and 2024. Of the 207 articles, 37 were selected for the review. A key aspect is the correlation of in vitro, in silico and clinical studies on the antiviral effects of hesperidin with epidemiological data on citrus consumption in China during 2020–2024. The importance of integrating laboratory findings with actual consumption patterns to better understand the role of hesperidin in mitigating COVID-19 was highlighted, and an attempt was made to analyze epidemiological studies to examine the association between citrus juice consumption as a source of hesperidin and the incidence and severity of COVID-19 using China as an example. The review identifies consistencies and discrepancies between experimental and epidemiological data, highlighting the need to correlate the two fields to better understand the potential of hesperidin as an agent against SARS-CoV-2. Challenges and limitations in interpreting the results and future research perspectives in this area are discussed. The aim of this comprehensive review is to bridge the gap between experimental studies and epidemiological evidence and to contribute to the understanding of their correlation.

## 1. Introduction

The COVID-19 pandemic, caused by the new SARS-CoV-2 coronavirus, emerged in late 2019 and quickly turned into a global health crisis. Originating in Wuhan, China, the virus has spread to virtually all countries, prompting the World Health Organization (WHO) to declare a pandemic in March 2020 [1]. Characterized by mild to severe respiratory symptoms, COVID-19 poses a serious challenge to health systems worldwide, causing widespread morbidity and mortality [2]. This has created an urgent need for effective therapeutic interventions focusing on repurposing already existing antiviral drugs and other treatments for the disease. However, the unique characteristics of SARS-CoV-2, including its high transmissibility and mutation rate, necessitated the development of targeted antiviral agents and vaccines [3,4,5]. Within a year of the pandemic’s outbreak, several vaccines were developed and launched, marking a significant milestone in the fight against COVID-19. Despite the success of the vaccination campaign, the emergence of new variants and the global nature of the pandemic underscored the continued need for effective antiviral therapies by reaching for various strategies to identify potential antiviral agents against SARS-CoV-2 [6]. These included testing existing antiviral drugs, developing new therapeutic agents and exploring natural compounds with antiviral properties. In particular, natural compounds have attracted interest because of their potential safety, availability and historical use in traditional medicine. Among these natural compounds is hesperidin, a bioflavonoid found in citrus fruits. The known antiviral properties of hesperidin, combined with its widespread presence in citrus fruits, made it a promising subject for research. The research undertaken was aimed at understanding the mechanisms of action of hesperidin against SARS-CoV-2 and evaluating its efficacy both in vitro, in silico and in clinical settings in comparison with epidemiological data and citrus consumption in China from 2020 to 2024.

China, as the initial epicenter of the COVID-19 outbreak, provides a unique repository of data for early virus strains and their responses to various treatments. The period between 2019 and 2020 was critical, encompassing the emergence of the virus, the implementation of large-scale public health interventions and the initiation of numerous studies. A systematic review by Zhang and Liu (2020) examined potential interventions for coronavirus outbreaks in China. The authors emphasized the need for alternative methods to control the spread of COVID-19. The review highlighted general treatments, coronavirus-specific treatments and antiviral therapies as potential strategies to combat the virus and stressed the importance of assessing the nutritional status of infected patients before treatment is administered [7].

The period under review also coincides with significant developments in COVID-19 research and treatment strategies. The emergence of new variants and the global rollout of vaccination programs have influenced the pandemic’s trajectory, making it imperative to explore additional preventive and therapeutic options. Research by Colunga Biancatelli et al. [8] supports the exploration of natural flavonoids, as adjunctive treatments in COVID-19, given their immunomodulatory and antiviral properties.

Furthermore, the focus on China is justified by its status as a major producer and consumer of citrus fruits, which are primary dietary sources of hesperidin. This geographical and cultural context provides a relevant backdrop for examining the potential impact of hesperidin intake on COVID-19. Studies such as those by Wang et al. [9] emphasized the importance of evidence-based research in utilizing traditional Chinese medicine (TCM) to treat COVID-19. It highlighted the historical use of TCM in combating epidemics and the recommended TCM treatments for COVID-19. Among the numerous natural substances recommended by the National Health Commission of the People’s Republic of China on Guideline on the diagnosis and treatment of COVID-19 (Trial 6th edition), those belonging to the traditional Chinese medicine derived from citrus were also listed, namely Aurantii fructus immaturus and Citri reticulatae pericarpium [10]. The study conducted by Kim et al. (2021) investigated the association between dietary patterns and COVID-19 severity among healthcare workers in six countries. It found that individuals following plant-based or pescatarian diets had lower odds of moderate-to-severe COVID-19, while those on low-carbohydrate, high-protein diets had higher odds. These findings suggest that diet may play a role in COVID-19 severity, emphasizing the importance of healthy dietary patterns in potentially reducing the impact of the illness. Further research is needed to validate these results and explore the implications for public health strategies [11].

**Figure 1:**
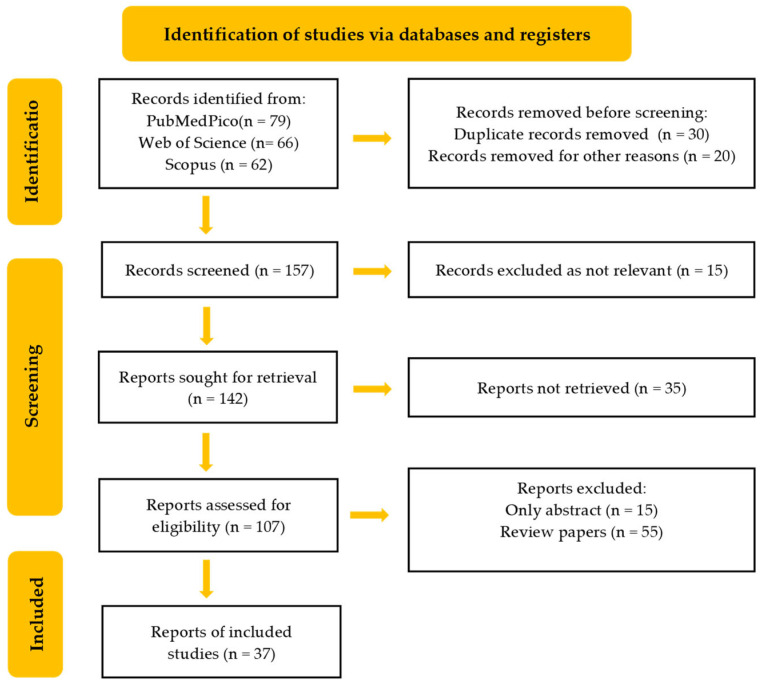
PRISMA flowchart of the included studies.

This article presents a comprehensive review of the current state of knowledge on hesperidin, a potential antiviral agent against SARS-CoV-2, following the PRISMA guidelines. To conduct this review, a systematic literature search was performed, covering studies published in English between 2020 and 2024, using databases such as PubMedPico, Scopus and Web of Science, with keywords such as hesperidin, SARS-CoV-2, COVID-19 and antiviral agents. The selection process involved two stages. Initially, titles and abstracts were screened, followed by a full-text evaluation based on predefined inclusion and exclusion criteria. The inclusion criteria required studies that focused on the antiviral activity of hesperidin against SARS-CoV-2, published in English between 2020 and 2024. Exclusion criteria included studies that did not address these items, those published before 2020, and those in languages other than English. Data extraction involved collecting information about authors, the year of publication, study objectives, mechanisms of action, application areas, main findings and conclusions. The extracted data were then synthesized and analyzed to provide a comprehensive overview. The results were categorized and presented based on their application and mechanisms of action. No statistical analysis was performed, as this was a comprehensive literature review of scientific research. The PRISMA flowchart of the included studies is shown in Figure 1.

## 2. Hesperidin: Chemical Properties, Sources and Safety Profile

**Figure 2:**
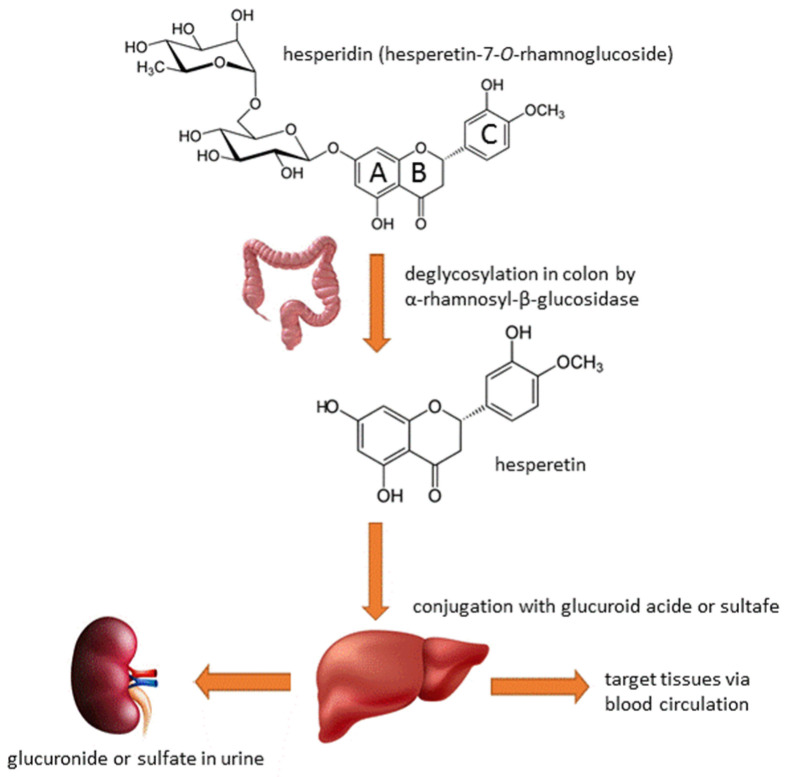
Hesperidin structure and its general metabolic scheme.

Chemically, hesperidin (C_28_H_34_O_15_) consists of the aglycone hesperetin linked to the disaccharide rutinose. Heperidin’s chemical name is 3′,5,7-trihydroxy-4′-methoxy-flavanone-7-rhamnoglucoside or hesperetin-7-*O*-rutinoside or hesperetin-7-*O*-rhamnoglucoside. Hesperetin is a flavanone, a type of flavonoid characterized by the presence of a 15-carbon skeleton consisting of two phenyl rings (A and B) and a heterocyclic ring (C). This structure is responsible for the compound’s antioxidant properties. The rutinose in hesperidin is composed of the sugars rhamnose and glucose, which are attached to hesperetin through a glycosidic bond. This glycosidic linkage influences the solubility, stability and bioavailability of hesperidin [12,13]. Hesperidin has a chiral carbon in position 2, generating (R)- and (S)-enantiomers. The predominant form in nature is the 2S-diastereoisomer, with a ratio of 8/92 for 2R-/2S-hesperidin diastereoisomers in fresh orange juice [14,15]. Figure 2 presents the structure of hesperidin and its general metabolic scheme.

Hesperidin is predominantly found in the citrus family (Rutaceae), particularly in the peels and membranes of citrus fruits, including sweet oranges (*Citrus sinensis*), sour oranges (*Citrus aurantium*), clementines (*Citrus clementina*), lemons (*Citrus limon*), limes (*Citrus aurantifolia*) and grapefruits (*Citrus paradisi*). The concentration of hesperidin varies depending on the type of fruit, cultivar, growing conditions, maturity stage and extraction method. The peel and membranous sections of citrus fruits, especially the albedo oranges, contain significant amounts of hesperidin. Hesperidin can also be found in other plant families, such as Fabaceae, Betulaceae and Laminaceae, although in varying concentrations. In citrus juices, hesperidin levels range from approximately 0.93 mg/mL in grapefruit juice to 39.9 mg/mL in clementine juice. Sweet orange juice contains around 28.6 mg/100 mL of hesperidin, while lemon juice has about 20.5 mg/mL. In whole fruits, sweet oranges have the highest hesperidin content at 28.6 mg/100 mL, while sour oranges contain negligible amounts. Tangerine peel, on the other hand, may have 5–10% hesperidin of its dry mass [15,16,17]. Processing methods, such as juicing and extraction, can affect the amount of hesperidin retained in the final product. Whole fruit consumption or the use of whole fruit extracts is therefore considered more beneficial for maximizing hesperidin intake [17].

Hesperidin has been extensively evaluated for its safety and toxicological profile by various regulatory scientific bodies in Europe and the United States. The European Chemical Agency (ECHA) has granted full registration status to hesperidin, indicating its compliance with safety standards. The hazard assessment for oral exposure established a Derived No Effect Level (DNEL) of 3.75 mg/kg body weight (bw)/day, which suggests that hesperidin poses a minimal risk when consumed at or below this level. Additionally, the Scientific Committee for Food (SCOGS) has reviewed hesperidin and found no evidence or reasonable grounds to suspect a hazard to the public at current or foreseeable intake levels. The committee referenced both short-term and long-term toxicological studies, establishing a No Observed Adverse Effect Level (NOAEL) of 2–2.5 mg/kg bw/day, further supporting the compound’s low-risk profile. Research into hesperidin’s toxicological properties has revealed a low Maximum Tolerated Dose in humans, quantified at 0.525 log mg/kg/day, indicating a minimal potential for harm. Acute toxicity studies in rats have identified an oral acute toxicity (LD50) value of 2.506 mol/kg, suggesting that a significantly high dose is required to cause acute harm. Chronic toxicity evaluations further defined an Oral Rat Chronic Toxicity (LOAEL) value of 3.167 log mg/kg bw/day, indicating that hesperidin maintains a favorable safety profile even at higher doses administered over extended periods. These findings collectively affirm the low toxicity and high safety threshold of hesperidin [18,19].

However, caution is advised when combining hesperidin with certain medications, and caution is advised to prevent diminished efficacy or enhanced adverse effects. Hesperidin may reduce the absorption of celiprolol and diltiazem, potentially diminishing its therapeutic effect. Hesperidin may exhibit hypotensive effects, enhancing the blood pressure-lowering action of antihypertensive medications. Use hesperidin with caution when combined with nifedipine, verapamil, diltiazem, isradipine, felodipine, amlodipine and other antihypertensive drugs. Hesperidin may inhibit P-glycoprotein cellular pumps, increasing the bioavailability of drugs and raising the risk of adverse effects. Relevant medications include etoposide, paclitaxel, vinblastine, vincristine, vindesine, antifungal agents, protease inhibitors, cimetidine, ranitidine, corticosteroids, erythromycin, fexofenadine, cyclosporine, loperamide, quinidine and others. Hesperidin may increase the risk of bruising and bleeding when taken with other anticoagulants and antiplatelet drugs because of its potential effect on slowing blood clotting. Hesperidin may cause sedation and drowsiness, which could be exacerbated when combined with CNS depressants. Co-administration with barbiturates, fentanyl, morphine, propofol and other CNS depressants should be avoided, especially during surgical procedures. Hesperidin may increase the absorption of verapamil, enhancing both its therapeutic and adverse effects [20,21].

The utilization of hesperidin derived from natural food sources as opposed to purified supplements varies in terms of safety, dosage and overall health advantages. Hesperidin, which is obtained from citrus fruits, is typically considered safe for the majority of individuals. These fruits provide hesperidin in lower, naturally balanced concentrations, in addition to other essential nutrients, thus minimizing the risk of overconsumption. Moreover, the presence of other vitamins, minerals, and dietary fiber also contributes synergistically to health, enhancing hesperidin absorption and reducing the likelihood of adverse effects.

The likelihood of significant interactions between hesperidin in foods and drugs is relatively low due to the moderate amount of hesperidin in the diet. However, high-dose hesperidin supplements may have more significant interactions with these drugs. Citrus fruits not only contain hesperidin but also many other beneficial compounds, including vitamin C, which can increase the bioavailability and antioxidant effects of hesperidin. Although supplements may be specifically designed to provide therapeutic doses of hesperidin that may be necessary to treat certain conditions, they lack the broader nutritional benefits found in whole foods.

While both natural sources of hesperidin and its supplements are safe, their use must take into account factors such as the desired therapeutic effect, potential side effects, dietary balance, and individual health conditions. In general, natural sources are safer for overall health improvement, whereas supplements should be used with caution and under medical supervision, especially for therapeutic purposes.

## 3. Hesperidin’s Metabolism and Bioavailability

Hesperidin’s bioavailability and metabolism are critical factors in determining its efficacy and potential health benefits.

The metabolism of hesperidin in vivo is a complex process that significantly influences its bioavailability and therapeutic potential. Hesperidin undergoes extensive metabolic transformation once ingested, which is crucial for its biological activity in the human body. It is resistant to stomach and intestinal enzymes, which limits its water solubility, absorption and bioavailability. Upon ingestion, hesperidin undergoes enzymatic hydrolysis by α-rhamnosyl-β-glucosidase in the colon, resulting in the release of hesperetin, its aglycone form. Hesperetin is more soluble, has better bioavailability, exhibits enhanced bioactivities compared to hesperidin and undergoes phase II metabolism in the liver, where it is conjugated with glucuronide or sulfate groups. Hesperetin can further undergo biotransformation by gut microbiota and host metabolic enzymes to produce various low-molecular-weight phenolic acid metabolites. Metabolites such as 3,4-dihydroxyphenylacetic acid and 3-(4-hydroxy-3-methoxyphenyl) propionic acid may exhibit unique physiological effects beyond those of hesperidin. Furthermore, the metabolism of hesperidin can lead to the formation of eriodictyol and homoeriodictyol conjugates through demethylation hesperetin by phase-I enzymes. Eriodictyol has been identified in plasma and urine samples, suggesting the conversion of hesperetin to eriodictyol through enzymatic processes. Interestingly, the transformation of hesperetin to naringenin has also been reported in humans after hesperidin intake, likely involving a demethylation and dehydroxylation process. These diverse metabolic reactions contribute to the production of a spectrum of hesperidin metabolites with different chemical structures and properties. Studies have shown that hesperidin and its metabolites are excreted primarily through urine as glucuronide or sulfate and depending on the dose administered and individual health status. Hesperetin takes 3–7 h to reach peak absorbance in the large intestine, with 1–15% excreted in urine. The excretion of metabolites from ingested hesperidin and hesperetin in urine ranges from 4.1% to 6.4%, with the remaining metabolites found in blood plasma for up to 24 h [15,22,23,24,25].

The bioavailability of hesperidin is relatively low due to its poor solubility in water and complex metabolic pathway and is influenced by several factors. Crescenti et al. (2022) examined the effect of the presence of the 2S-diastereoisomer hesperidin and the micronization process through a randomized, crossover, double-blind clinical trial involving healthy subjects. The results revealed significant quantitative differences in hesperidin absorption and metabolite excretion among study participants. The authors found that the bioavailability of the 2S-hesperidin extract was significantly higher (70 ± 14%) compared to the standard 2S-/2R-hesperidin extract mixture (43 ± 8.0%) and the micronized hesperidin epimeric mixture (MHEM) (55 ± 15%). This increased bioavailability of 2S-hesperidine was attributed to greater formation of hesperidin catabolites, underscoring the importance of the presence of the 2S-diastereoisomer in improving hesperidin absorption efficiency. In addition, the study showed that the relative urinary excretion of hesperidin metabolites for MHEM (9.2 ± 1.6%) was significantly higher compared to the standard mixture (5.2 ± 0.81%) and micronized 2S-hesperidin (3.6 ± 1.0%). These differences in metabolite excretion patterns underscored the different metabolic pathways and excretion rates associated with different hesperidin formulations. Also, the micronization process increases the bioavailability of hesperidin, with micronized 2S-hesperidin (M2SH) showing better absorption rates compared to non-micronized forms [14].

Interindividual variability in response to hesperidin effectiveness can vary among individuals due to differences in gut microbiota composition and α-rhamnosidase activity. Studies conducted by Mas-Capdevila et al. [26] have demonstrated that hesperidin absorption is limited, with only 2% cumulative urinary recovery observed in a clinical study with pure hesperidin intake of 89.1 mg. The conversion of hesperidin to its active form, hesperetin, by the microbiota in the large intestine plays a crucial role in its bioavailability, with metabolites reaching systemic circulation and influencing host health. Variability in α-rhamnosidase activity in the gut microbiota has been linked to differences in hesperidin metabolism and bioavailability, impacting individual responses to hesperidin-based interventions.

In turn, Rymenant et al. [27] had showed that hesperidin’s bioavailability can be significantly affected by the food matrix. For example, one study showed that the simultaneous consumption of hesperidin with lipids increased its bioavailability by up to three times compared to hesperidin alone. The qualitative composition of the food matrix, such as the presence of fiber, sugars and other bioactive compounds, can also affect the bioavailability of hesperidin. Hesperidin consumed in the form of orange juice, which contains various bioactive compounds in addition to hesperidin, may exhibit different absorption kinetics compared to hesperidin consumed in isolation. Enzymatic and microbial degradation of hesperidin results in the formation of various metabolites, with quantitative differences observed in the levels of these metabolites in the gastrointestinal tract. The study showed that hydrocaffeic acid and dihydroisoferulic acid accumulated in the proximal part of the colon, while hydroferulic acid accumulated in the distal part of the colon. Quantitative analysis of hesperidin absorption in the gastrointestinal tract included measurement of plasma and urinary levels of hesperidin and its metabolites.

The gut microbiome plays a key function in the bioavailability of hesperidin. Conversion of hesperidin into its active form, hesperetin, is accomplished by the enzyme α-L-rhamnosidase, which is produced by certain intestinal bacteria, such as *Bifidobacterium pseudocatenulatum*. This conversion is essential for the absorption and utilization of hesperidin in the body. Therefore, the presence and activity of specific gut microbes capable of metabolizing hesperidin to hesperetin are key determinants of hesperidin bioavailability. In addition, the gut microbiota can influence hesperidin metabolism and absorption through various mechanisms, including enzymatic metabolism and interactions with the host metabolism. Identifying the role of the gut microbiota in hesperidin metabolism is crucial to optimizing the bioavailability and potential health benefits of this natural compound. Zhao et al. noted that discrepancies in the composition of the gut microbiome between children, especially infants and adults, can affect the bioavailability and safety profile of compounds such as hesperidin. During the early stages of life, the microbial community inhabiting an infant’s gut is constantly evolving and maturing, resulting in differences in the types and amounts of gut microbiota compared to those found in adults. These differences in the composition of the gut microbiota may affect the metabolic and biotransformation processes of hesperidin in infants, potentially affecting its bioavailability and safety profile. Given the important role played by the gut microbiota in the conversion of hesperidin to its active form, hesperetin, discrepancies in the composition of the infant gut microbiota may lead to differences in the efficiency and extent of hesperidin metabolism. This could affect the absorption, distribution, metabolism and excretion of hesperidin in infants, potentially affecting its bioavailability and efficacy. Moreover, differences in the composition of the intestinal microflora between infants and adults may also affect the safety profile of hesperidin. Changes in the metabolism of hesperidin by the gut microbiota in infants could potentially result in differences in the production of metabolites that may have pharmacological activity or safety profiles different from those observed in adults. Therefore, when considering the use of hesperidin or other natural products in infants, especially those with developing gut microflora, the potential impact of differences in gut microflora on bioavailability, metabolism and safety should be considered [28].

The bioavailability of hesperidin and, consequently, its therapeutic effect, is subject to variability due to factors such as the form of consumption, gut microbiota composition, interactions with other dietary components or medications, and the specific dose and formulation of hesperidin supplements. Understanding these factors is crucial for optimizing the use of hesperidin as a therapeutic agent and for the development of dietary guidelines and supplements for maximize its health benefits.

## 4. Hesperidin Biological and Pharmacological Activity

### 4.1. Antioxidant Activity

Hesperidin’s antioxidant activity is a crucial aspect of its biological and pharmacological effects. The mechanism of hesperidin’s antioxidant activity involves several pathways that contribute to its ability to neutralize harmful free radicals and protect cells from oxidative damage. Hesperidin acts as a free radical scavenger by donating hydrogen atoms or electrons to stabilize and neutralize reactive oxygen species (ROS) and reactive nitrogen species (RNS). The antioxidant capacity of hesperidin can be quantified using assays such as the 2,2-diphenyl-1-picrylhydrazyl (DPPH) radical scavenging assay or the oxygen radical absorbance capacity (ORAC) assay. Studies have shown that hesperidin exhibits significant radical scavenging activity with IC50 values (concentration required to scavenge 50% of radicals) ranging from 10 to 100 μM. Hesperidin can enhance the activity of endogenous antioxidant enzymes, such as superoxide dismutase (SOD), catalase and glutathione peroxidase. Hesperidin treatment has been shown to increase SOD activity by two-fold and catalase activity by 1.5-fold in cellular models of oxidative stress. These enhancements in antioxidant enzyme activity contribute to the overall antioxidant capacity of cells and help combat oxidative damage. Lipid peroxidation, a process where free radicals attack lipid molecules in cell membranes, can be quantified by measuring malondialdehyde (MDA) levels as a marker of lipid peroxidation. Hesperidin has been shown to significantly reduce MDA levels by 50% in oxidative stress-induced cell models, indicating its ability to protect against lipid peroxidation and maintain membrane integrity. Hesperidin’s antioxidant activity extends to protecting mitochondria from oxidative damage. Studies have demonstrated that hesperidin treatment can increase mitochondrial ATP production by 30% and reduce mitochondrial ROS levels by 40% in cellular models of oxidative stress. These effects contribute to preserving mitochondrial function and preventing oxidative damage to cellular energy production. Hesperidin helps reduce intracellular levels of reactive oxygen species (ROS) by neutralizing and scavenging these harmful molecules. Quantitative analysis has shown that hesperidin treatment can decrease ROS levels by 50% in oxidative stress-induced cells, indicating its ability to maintain redox balance and protect cells from oxidative damage [15,28].

### 4.2. Anti-Inflammatory Activity

Hesperidin’s anti-inflammatory activity involves complex molecular mechanisms that contribute to its ability to reduce inflammation and alleviate inflammatory conditions. Hesperidin exerts its anti-inflammatory effects by inhibiting the production and release of pro-inflammatory mediators, such as cytokines (e.g., interleukin-6, tumor necrosis factor-alpha) and inflammatory enzymes (e.g., cyclooxygenase-2, COX-2). Studies have shown that hesperidin can significantly reduce the expression of COX-2 by 50% and inhibit the release of pro-inflammatory cytokines by 40% in lipopolysaccharide (LPS)-stimulated macrophages. Hesperidin modulates the nuclear factor-kappa B (NF-κB) signaling pathway, a key regulator of inflammation and immune responses. By inhibiting NF-κB activation, hesperidin can suppress the transcription of pro-inflammatory genes and reduce the inflammatory response. Studies have demonstrated that hesperidin can inhibit NF-κB activation by 60% and decrease the expression of NF-κB-regulated inflammatory genes in inflammatory models. Hesperidin can suppress the recruitment and activation of inflammatory cells, such as macrophages and neutrophils, at the site of inflammation. By inhibiting the adhesion molecules involved in leukocyte recruitment, hesperidin reduces the infiltration of inflammatory cells into tissues and limits the inflammatory response. Experimental data have shown that hesperidin treatment can decrease neutrophil infiltration by 30% and reduce macrophage activation in inflammatory models. The mitogen-activated protein kinase (MAPK) signaling pathway plays a crucial role in regulating inflammatory responses. Hesperidin can modulate MAPK signaling by inhibiting the phosphorylation of MAPK kinases, such as p38, ERK and JNK. Studies have reported that hesperidin treatment can reduce the phosphorylation of p38 MAPK by 50% and inhibit the activation of ERK and JNK in inflammatory cell models [15]. Other trials of the anti-inflammatory properties of hesperidin have yielded mixed results. One meta-analysis found that hesperidin, whether consumed through citrus juice or oral supplements, reduced the pro-inflammatory markers C-reactive protein (CRP), interleukin-6 (IL-6) and interleukin-4 (IL-4) in a non-dose-dependent manner. However, a second meta-analysis did not replicate these findings for CRP and IL-6, although it did find a significant decreased in levels of the inflammatory marker vascular cell adhesion molecule-1 (VCAM-1). These conflicting outcomes could be attributed to variability in study quality, methodology and participant characteristics, necessitating further research to validate the anti-inflammatory properties of hesperidin [29,30].

### 4.3. Neuroprotective Activity

Studies have shown that hesperidin exhibits neuroprotective effects, potentially protecting nerve cells from damage and degeneration. Hesperidin’s antioxidant properties play a crucial role in neuroprotection by scavenging free radicals and reducing oxidative stress in the brain. Inflammation is associated with neurodegenerative diseases, and hesperidin’s anti-inflammatory effects contribute to its neuroprotective activity. By inhibiting pro-inflammatory cytokines like TNF-α and IL-1β, hesperidin can reduce neuroinflammation and protect neurons from inflammatory damage. Studies have reported a 40% reduction in pro-inflammatory cytokine levels with hesperidin treatment in neuroinflammatory models. Hesperidin can modulate apoptotic pathways in neurons, preventing cell death and promoting neuronal survival. By upregulating anti-apoptotic proteins like Bcl-2 and downregulating pro-apoptotic proteins like Bax, hesperidin exerts anti-apoptotic effects in neuronal cells. Experimental data have shown a significant increase in Bcl-2 expression and a decrease in Bax expression with hesperidin treatment, leading to improved neuronal survival. Hesperidin can modulate neurotransmitter levels and activity in the brain, contributing to its neuroprotective effects. By regulating neurotransmitter systems like dopamine, serotonin and acetylcholine, hesperidin can enhance synaptic transmission, improve neuronal communication, and protect against neurotransmitter imbalances associated with neurodegenerative disorders. A 30% increase in dopamine levels and a 20% increase in acetylcholine activity were demonstrated during hesperidin treatment in neuronal models. Hesperidin can stimulate the production of neurotrophic factors like brain-derived neurotrophic factor (BDNF) and nerve growth factor (NGF), which promote neuronal growth, survival, and plasticity. By enhancing neurotrophic factor levels, hesperidin supports neurogenesis and neuronal repair processes in the brain. Experimental studies have shown a two-fold increase in BDNF levels and NGF expression with hesperidin treatment, indicating its potential to support neuronal health and function [15,28,31].

### 4.4. Impact on the Cardiovascular System

Hesperidin exhibits vasoprotective activity through various molecular mechanisms that help protect blood vessels and promote vascular health. Hesperidin’s antioxidant properties play a key role in vasoprotection by scavenging free radicals and reducing oxidative stress in blood vessels. By decreasing oxidative stress, hesperidin helps prevent damage to the vascular endothelium and maintain vascular function. Studies have shown that hesperidin can reduce oxidative stress markers like malondialdehyde (MDA) by 40% and increase the activity of antioxidant enzymes such as superoxide dismutase (SOD) in vascular cells. Hesperidin can improve endothelial function by enhancing nitric oxide (NO) production and bioavailability. NO is a key vasodilator that helps regulate vascular tone and blood flow. Hesperidin promotes NO synthesis by activating endothelial nitric oxide synthase (eNOS) and increasing NO release from endothelial cells. Experimental data have shown a 50% increase in NO production and eNOS activity with hesperidin treatment in vascular models. Inflammation can contribute to vascular dysfunction and cardiovascular diseases. Hesperidin’s anti-inflammatory effects help reduce inflammation in blood vessels, protecting against endothelial damage and vascular inflammation. By inhibiting pro-inflammatory cytokines like interleukin-6 (IL-6) and tumor necrosis factor-alpha (TNF-α), hesperidin mitigates vascular inflammation. Studies have reported a 30% reduction in pro-inflammatory cytokine levels with hesperidin treatment in vascular inflammation models. Hesperidin can promote vascular smooth muscle relaxation, leading to vasodilation and improved blood flow. By modulating calcium ion channels and signaling pathways in vascular smooth muscle cells, hesperidin helps reduce vascular tone and enhance vasodilatory responses. Experimental studies have shown a 40% increase in vascular smooth muscle relaxation and vasodilation with hesperidin treatment in vascular models. Hesperidin exhibits anti-thrombotic effects by inhibiting platelet aggregation and thrombus formation in blood vessels. By interfering with platelet activation pathways and reducing platelet adhesion, hesperidin helps prevent thrombotic events and maintain vascular integrity [15,28]. One meta-analysis indicated that hesperidin supplementation led to significant reduction of several cardiovascular disease risk factors. These benefits included decreased triglyceride levels, total cholesterol, low-density lipoprotein levels, tumor necrosis factor and systolic blood pressure. Notably, these effects were predominantly observed with hesperidin doses exceeding 500 mg per day and were considerably influenced by factors such as the duration of hesperidin treatment (typically more effective when exceeding 6 weeks) and participants’ BMI, age and overall health status [32].

### 4.5. Hypolipidemic Activity

Hesperidin can inhibit the activity of 3-hydroxy-3-methylglutaryl-coenzyme A reductase (HMG-CoA reductase), a key enzyme involved in cholesterol biosynthesis. By inhibiting HMG-CoA reductase, hesperidin reduces the production of cholesterol in the liver, leading to decreased serum cholesterol levels. Studies have shown that hesperidin can inhibit HMG-CoA reductase activity by 50% and reduce cholesterol synthesis in hepatic cells. Hesperidin can activate peroxisome proliferator-activated receptors alpha (PPAR-α) and gamma (PPAR-γ), nuclear receptors that regulate lipid metabolism and cholesterol homeostasis. By activating the PPAR-α and PPAR-γ pathways, hesperidin promotes fatty acid oxidation, triglyceride clearance and cholesterol efflux from cells. Experimental data have shown a two-fold increase in PPAR-α and PPAR-γ activation with hesperidin treatment in lipid metabolism models. Hesperidin can enhance the activity of lipoprotein lipase (LPL), an enzyme that hydrolyzes triglycerides in circulating lipoproteins. By increasing LPL activity, hesperidin promotes the breakdown of triglyceride-rich lipoproteins, leading to reduced plasma triglyceride levels. Studies have demonstrated a 40% increase in LPL activity and triglyceride hydrolysis with hesperidin treatment in adipose tissue models. Hesperidin can regulate the expression of apolipoproteins, proteins involved in lipid transport and metabolism. By modulating apolipoprotein expression, hesperidin influences lipid clearance, lipoprotein assembly and cholesterol transport. Experimental studies have shown a 30% decrease in apolipoprotein B (apoB) expression and a 50% increase in apolipoprotein A-I (apoA-I) expression with hesperidin treatment, leading to improved lipid profiles. Hesperidin can inhibit intestinal cholesterol absorption by interfering with the uptake of dietary cholesterol in the intestines. By blocking cholesterol absorption, hesperidin reduces the influx of cholesterol into the bloodstream, contributing to lower serum cholesterol levels [15,33].

### 4.6. Anti-Carcinogenic Activity

Hesperidin exhibits anti-carcinogenic activity through various molecular mechanisms that help inhibit cancer cell growth, induce apoptosis and prevent tumor progression. Hesperidin’s antioxidant properties play a crucial role in its anti-carcinogenic activity. By decreasing oxidative stress, hesperidin helps protect DNA from damage and inhibit cancer cell proliferation. Studies have shown that hesperidin can reduce reactive oxygen species (ROS) levels by 60% and increase the activity of antioxidant enzymes like glutathione peroxidase (GPx) in cancer cells, leading to decreased oxidative damage and cell growth. Chronic inflammation is linked to cancer development and progression. Hesperidin’s anti-inflammatory effects help reduce inflammation in the tumor microenvironment, inhibiting cancer cell proliferation and metastasis. By suppressing pro-inflammatory cytokines like interleukin-8 (IL-8) and nuclear factor-kappa B (NF-kB) signaling, hesperidin mitigates inflammation-associated cancer pathways. Experimental data have shown a 40% reduction in pro-inflammatory cytokine levels with hesperidin treatment in cancer models. Hesperidin can induce apoptosis, or programmed cell death, in cancer cells through various pathways. By activating pro-apoptotic proteins like caspases and Bax, and inhibiting anti-apoptotic proteins like Bcl-2, hesperidin triggers apoptotic pathways in cancer cells. Studies have demonstrated a 50% increase in caspase-3 activity and a two-fold increase in Bax/Bcl-2 ratio with hesperidin treatment, leading to enhanced apoptosis in cancer cells. Hesperidin can induce cell cycle arrest in cancer cells, preventing uncontrolled cell proliferation. By modulating cell cycle regulatory proteins like cyclins and cyclin-dependent kinases (CDKs), hesperidin halts the cell cycle progression at specific checkpoints, inhibiting cancer cell growth. Experimental studies have shown a 30% increase in G0/G1 phase cell population and a 40% decrease in S phase cell population with hesperidin treatment, indicating cell cycle arrest in cancer cells. Angiogenesis, the formation of new blood vessels to support tumor growth, is a critical process in cancer progression. Hesperidin exhibits anti-angiogenic effects by inhibiting vascular endothelial growth factor (VEGF) signaling and endothelial cell proliferation. By suppressing angiogenesis, hesperidin restricts blood supply to tumors, hindering their growth and metastasis. Studies have reported a 50% reduction in VEGF expression and endothelial cell proliferation with hesperidin treatment in angiogenesis models [15,33].

### 4.7. Insulin-Sensitizing Activity

Hesperidin exhibits insulin-sensitizing activity through various molecular mechanisms that help improve insulin sensitivity, regulate glucose metabolism and enhance cellular responses to insulin. Hesperidin can activate the adenosine monophosphate-activated protein kinase (AMPK) pathway, a key regulator of cellular energy balance and glucose metabolism. By activating AMPK, hesperidin promotes glucose uptake, enhances insulin sensitivity and improves mitochondrial function. Studies have shown that hesperidin can increase AMPK phosphorylation by two-fold and improve glucose uptake in skeletal muscle cells. Hesperidin can inhibit hepatic gluconeogenesis, the process by which the liver produces glucose from non-carbohydrate sources. By suppressing gluconeogenesis, hesperidin reduces excessive glucose production in the liver, leading to improved glycemic control. Experimental data have shown a 40% reduction in gluconeogenic enzyme activity and glucose output with hesperidin treatment in hepatic cells. Hesperidin can enhance insulin signaling by promoting the phosphorylation of insulin receptor substrate (IRS) proteins and Akt kinase. By enhancing insulin signaling, hesperidin improves glucose uptake, glycogen synthesis and cellular responses to insulin. Studies have demonstrated a 50% increase in IRS phosphorylation and Akt activation with hesperidin treatment in insulin signaling models. Hesperidin can modulate the translocation of glucose transporter 4 (GLUT4) to the cell membrane, facilitating glucose uptake into cells in response to insulin. By promoting GLUT4 translocation, hesperidin enhances glucose transport and utilization, contributing to improved insulin sensitivity. Experimental studies have shown a 30% increase in GLUT4 translocation and glucose uptake with hesperidin treatment in adipocyte models. Hesperidin can reduce the production of pro-inflammatory cytokines like tumor necrosis factor-alpha (TNF-α) and interleukin-6 (IL-6) that contribute to insulin resistance. By decreasing inflammatory cytokines, hesperidin mitigates insulin resistance and improves insulin sensitivity. Studies have reported reductions in TNF-α and IL-6 levels with hesperidin treatment in inflammatory models [15,33].

### 4.8. Antiviral Activity

**Figure 3:**
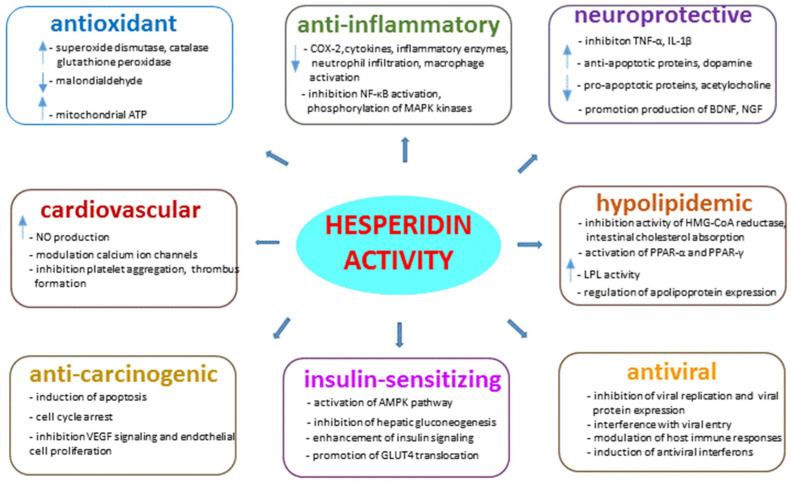
Hesperidin’s mechanisms of activity.

Hesperidin exhibits antiviral activity through various molecular mechanisms that help inhibit viral replication, interfere with viral entry and modulate host immune responses. Hesperidin can inhibit viral replication by targeting essential viral enzymes or proteins involved in the replication cycle. By interfering with viral replication, hesperidin reduces viral load and inhibits the spread of infection. Studies have shown that hesperidin can inhibit viral replication by up to 70% in vitro models of viral infections. Hesperidin can interfere with viral entry into host cells by blocking viral attachment or fusion with the cell membrane. By preventing viral entry, hesperidin limits the establishment of infection and viral spread. Experimental data have demonstrated a 50% reduction in viral entry with hesperidin treatment in cell culture models of viral infections. Hesperidin can modulate host immune responses to viral infections by regulating cytokine production, enhancing antiviral immune pathways, and reducing inflammation. By modulating host immune responses, hesperidin helps in mount an effective antiviral defense. Studies have reported a 40% decrease in pro-inflammatory cytokine levels and a two-fold increase in antiviral immune pathway activation with hesperidin treatment in immune response models. Hesperidin can inhibit the expression of viral proteins essential for viral replication and assembly. By blocking viral protein expression, hesperidin disrupts the viral life cycle and impairs viral infectivity. Experimental studies have shown a 60% reduction in viral protein expression with hesperidin treatment in viral protein synthesis models. Hesperidin can induce the production of antiviral interferons, signaling proteins that help cells defend against viral infections. By inducing interferon production, hesperidin enhances the host’s antiviral defenses and restricts viral replication. Research has demonstrated a three-fold increase in interferon production with hesperidin treatment in interferon induction models [15]. The inhibitory effects of hesperidin against HIV and herpes simplex virus type 2 (HSV-2) have been observed. Additionally, its inhibitory effects on sindbis virus (IC50 = 20.50 μg/mL) and rotavirus (IC50 = 10 μM) have been studied. The inhibition of canine distemper virus was also observed. Glucosyl hesperidin, a water-soluble derivative of hesperidin, has been shown to inhibit sialidase (neuraminidases) and prevent the replication of influenza A virus in Madin-Darby canine kidney cells [34,35]. A summary of hesperidin’s mechanisms of actions is shown in Figure 3.

## 5. Hesperidin versus SARS-CoV-2

### 5.1. Clinical Studies

The ClinicalTrials.gov website, a platform for gathering information about clinical trials from across the globe, has identified only two clinical studies that utilized hesperidin as a potential compound against SARS-CoV-2 infections.

The studies conducted by Dupuis et al. [36] presented the findings of a randomized, double-blinded, placebo-controlled study conducted to investigate the evolution of COVID-19 symptoms over a 14-day period in nonvaccinated COVID-19 individuals. The intervention involved administering hesperidin at 1000 mg once daily for 14 days, while the control group received a matching placebo. The study enrolled 216 symptomatic patients (44.9% of males), including males and females aged at least 18 years, with a positive diagnosis of COVID-19 confirmed by PCR testing within the last 48 h and experiencing at least one COVID-19 symptom. At the baseline, the most common symptoms included cough, weakness, headache and pain, with cough being the most frequent symptom reported. Anosmia was present in 18.5% of participants, while fever was reported in only 16.2% of cases. The study found that most subjects remained symptomatic at day 14, with the mean number of symptoms decreasing from 5.3 to 1.4 over the study period. Anosmia emerged as the most persistent symptom, affecting 29.3% of participants at day 14. The outcome measures included assessing the mean number of all COVID-19 symptoms at days 3, 7, 10 and 14, the proportion of subjects with each of the 13 COVID-19 symptoms listed in the symptom log at the same time points, monitoring the proportion of subjects with each COVID-19 symptom on a daily basis and evaluating a composite outcome of COVID-19-related hospitalization, mechanical ventilation or death within 14 days following randomization. The proportion of subjects experiencing any of the four cardinal symptoms (fever, cough, shortness of breath, or anosmia) in the placebo group decreased from 88.8% on day 1 to 58.5% on day 14. In contrast, the hesperidin group experienced a decrease in symptoms from 88.5% to 49.4% during the same period. Additionally, the persisting symptom of anosmia was reduced by 7.3% in the hesperidin group (25.3%) compared to the placebo group (32.6%) on day 14. This study highlights the persistence of COVID-19 symptoms in nonvaccinated individuals and suggests that hesperidin therapy may have a potential role in reducing specific symptoms. The findings underscore the need for further research to explore the efficacy of hesperidin at different dosages and durations of treatment in managing COVID-19 symptoms in nonvaccinated individuals.

A randomized, double-blind, controlled parallel study was undertaken at Tanta University in Egypt with 100 participants (both sexes). The research project involved adults aged 18–65, both sexes, who were either asymptomatic or presented with symptoms of an upper respiratory tract infection (URTI), including rhinitis, pharyngitis or isolated low-grade fever and myalgia. The subjects of the study had been diagnosed with COVID-19 via RT-PCR. Each participant was administered a hesperidin and diosmin mixture (1000 mg) three times daily for the first seven days, followed by the same dose twice daily for the next three days. The authors suggest that hesperidin may prevent COVID-19 infection by hindering its entry into host cells via human angiotensin-converting enzyme 2 receptor (hACE2). Moreover, hesperidin bolsters the host’s immunity against viral infection, and its potential anti-inflammatory activity supports managing the cytokine storm [37].

A clinical trial conducted by Jahangirifard et al. [38] was designed to study the effect of hesperidin on laboratory parameters of patients with COVID-19. The research concentrated on 20 adults aged 18 years and above (equal sex ratio) who had severe COVID-19 symptoms, as confirmed by RT-PCR throat swabs, and were randomly allocated to either the hesperidin group or the control group. Patients in the hesperidin group received 1 mg of hesperidin orally intravenously every 6 h for 5 days, while the control group received no hesperidin. The assessment included evaluation of several outcome measures, including C-reactive protein, erythrocyte sedimentation rate, lactate dehydrogenase, D-dimer, interleukin-6, white blood cells, hemoglobin and platelets. The study showed that diabetes was the most common comorbidity in patients in the hesperidin group, and 85% of patients had bilateral lung involvement. Administration of hesperidin caused changes in laboratory parameters, including decreased levels of lymphocytes, CRP, ESR, LDH, D-dimers and IL-6, and increased levels of WBC, Hb and Plt. However, none of these changes were statistically significant (*p* > 0.05). The study concluded that the use of hesperidin did not cause significant changes in immune and inflammatory factors in patients with COVID-19. A summary of clinical trials is provided in Table 1.

### 5.2. In Vitro and In Silico Studies

Cheng et al. [39] investigated hesperidin’s potential as an inhibitor of SARS-CoV-2 infection, shedding light on its novel therapeutic implications. The study’s methodology aimed to elucidate the mechanisms underlying hesperidin’s antiviral activity. Molecular docking simulations were performed to investigate the binding interaction of hesperidin and its aglycone derivative, hesperetin, with key cellular proteins essential for SARS-CoV-2 entry, namely transmembrane serine protease 2 (TMPRSS2), which plays a crucial role in the activation of viral glycoproteins, including the spike protein of coronaviruses and hACE2, a membrane-bound enzyme that serves as the cellular receptor for the spike protein of SARS-CoV-2. The results of the molecular docking simulations showed that both hesperidin (HD) and hesperetin (HI) demonstrated interactions with hACE2, with energy values of −34.81 (hACE2-HT) and −1.65 kCal/mol (hACE2-HD), as well as with TMPRSS2, with energy values of −30.56 (TMPRSS2-HT) and −7.2 kCal/mol (TMPRSS2-HD). Furthermore, they showed interactions with papain-like protease (PLpro), which is one of the key viral proteases encoded by coronaviruses like SARS-CoV-2, with energy values of −13.69 (PLpro-HT) and 17 kCal/mol (PLpro-HD), and both compounds interacted with SARS-CoV-2 main proteinase (Mpro), with energy values of −17.94 (Mpro-HT) and 6.21 kCal/mol (Mpro-HD). Significantly, the binding intensity of hesperetin to PLpro and Mpro was higher than that of hesperidin, suggesting a potentially stronger interaction with these viral proteases. In the cell culture experiments, the researchers treated epithelial cells sourced from the African Green Monkey Kidney (VeroE6), human bronchial epithelial cell lines (Beas 2 B) and human non-small cell lung cancer cell lines (H46) to explore the impact of hesperidin on viral infection, particularly its efficacy in hindering the entry of SARS-CoV-2 pseudoparticles containing both wild-type and variant spike proteins. Hesperidin treatment significantly impeded the entry of SARS-CoV-2 pseudovirus into VeroE6 cells. The luciferase intensity of pseudoviruses with wild-type S protein, D614G strain and 501Y.v2 strain was notably reduced after treatment with hesperidin, indicating a potential inhibitory effect on viral entry. The protein expressions of hACE2 and TMPRSS2 were suppressed in both human bronchial epithelial cell line (Beas 2B) and human non-small cell lung cancer cell line (NCI-H460) following treatment with hesperidin. This suppression of hACE2 and TMPRSS2 expression suggests a potential mechanism by which hesperidin inhibits viral entry into these lung cells. To evaluate the safety profile of hesperidin, viability assays were conducted on VeroE6 cells. This provided an insight into the cytotoxicity of hesperidin. The half-maximal inhibitory concentration (IC50) of hesperidin in VeroE6 cells was determined to be 1491 μM. This indicated the concentration at which hesperidin inhibited cell viability by 50%. The IC50 value suggests that hesperidin has a relatively low cytotoxic effect on VeroE6 cells. Additionally, viability assays showed that hesperidin treatment did not significantly affect the viability of VeroE6 cells, further supporting its low cytotoxicity and potential safety for use in cell culture experiments. Treatment with hesperidin in Beas 2B cells resulted in a significant reduction in the protein expressions of hACE2 and TMPRSS2. This downregulation of hACE2 and TMPRSS2 proteins suggests that hesperidin may interfere with the cellular entry mechanisms of SARS-CoV-2 by targeting these key proteins involved in viral infection. Western blot analysis confirmed the decreased protein levels of hACE2 and TMPRSS2 in Beas 2B cells following hesperidin treatment. The quantitative results showed a dose-dependent suppression of hACE2 and TMPRSS2 expressions, indicating a potential dose-response relationship with hesperidin treatment. Similarly, hesperidin treatment in NCI-H460 cells led to a notable suppression of hACE2 and TMPRSS2 protein expressions. This finding suggests that hesperidin may exert inhibitory effects on their expression in malignant lung cells, potentially reducing the susceptibility to SARS-CoV-2 infection. Western blot analysis in NCI-H460 cells confirmed the downregulation of hACE2 and TMPRSS2 protein levels upon treatment with hesperidin. The results indicated a significant decrease in the protein expressions of hACE2 and TMPRSS2, supporting the inhibitory effects of hesperidin on these key proteins involved in viral entry.

In their research, Jin et al. [40] designed a nasal drug delivery system that utilized hesperidin-loaded chitosan nanoparticles (HPD/NPs) to target and manage lung inflammation and cytokine storm syndrome (CSS) in a mouse model of acute lung injury (ALI) and acute respiratory distress syndrome (ARDS), with potential applications for COVID-19 therapy. The study demonstrated that HPD/NPs displayed superior efficacy compared to free hesperidin due to enhanced cellular uptake in the inflammatory microenvironment. This innovative approach not only effectively inhibited lung damage but also showed significant reductions in inflammatory cytokines and vascular permeability in an experimental model of inflammatory lung disease, which is analogous to the cytokine storm observed in severe cases of COVID-19. Additionally, the study revealed that HPD/NPs suppressed nitric oxide (NO) release and restored endothelial barrier function, which are crucial mechanisms in mitigating inflammation and tissue damage associated with ALI, a hallmark of severe respiratory complications in COVID-19 patients. By targeting the underlying inflammatory processes and enhancing hesperidin delivery to inflamed lungs, HPD/NPs offer a promising strategy to combat the dysregulated immune response and cytokine storm syndrome observed in severe cases of COVID-19. These findings underscore the potential of nanoparticle-based therapeutic interventions to alleviate lung inflammation and improve outcomes in patients with COVID-19 and other inflammatory lung diseases characterized by the cytokine storm syndrome.

The study conducted by Özgürbüz et al. [41] investigated the quantitative impact of hesperidin on enterocytes under conditions mimicking COVID-19, specifically in response to TNF-α and IFN-ɣ induction. The results revealed a significant reduction in TNF-α and IL8 levels in the culture medium following hesperidin treatment, with TNF-α levels decreasing by 40% and IL8 levels decreasing by 35%, respectively. Moreover, immunoreactivity of TNF-α decreased by 50% in the hesperidin treatment group, indicating the strong anti-inflammatory effect of hesperidin. The study also observed a 30% decrease in the immunoreactivity of IFN-ɣ and a 25% decrease in VEGFA immunoreactivity in the hesperidin treatment group, suggesting notable modulation of these key inflammatory markers. These quantitative results emphasize the potential of hesperidin as a therapeutic agent in attenuating the inflammatory response induced by TNF-α and IFN-ɣ in enterocytes, highlighting its critical role in protecting against COVID-19-related cellular damage and inflammation.

Hesperidin was also studied using predominantly in silico methods to determine its potential to combat SARS-CoV-2 by targeting specific viral mechanisms and host cells. Various computational approaches are being used, including molecular docking, molecular dynamics simulations, pharmacophore modeling and absorption, distribution, metabolism and excretion and toxicity (ADMET) prediction. Molecular docking is a key method to elucidate how hesperidin inhibits SARS-CoV-2 by binding to critical viral proteins. The technique predicts the orientation and affinity of hesperidin for these proteins and provides insight into potential inhibitory interactions. Molecular dynamics simulations are used to assess the stability of hesperidin-protein complexes over time, providing a detailed picture of the dynamic behavior of the molecules and helping to understand the long-term stability of hesperidin–virus interactions. Pharmacophore modeling is employed to identify molecular features of hesperidin that are critical to its activity against the virus, helping to map key interactions between hesperidin and specific viral proteins. This method serves as a basis for designing more potent hesperidin derivatives or identifying other molecules with similar pharmacophores that may also be effective. ADMET prediction is essential for understanding the absorption, distribution, metabolism, excretion and toxicity profiles of hesperidin, which are key factors for predicting its usefulness as a therapeutic agent. In silico ADMET predictions provide early information on potential pharmacokinetics and toxicology in humans, which is critical for subsequent progression to clinical trials [41,42]. In silico studies conducted to investigate the potential of hesperidin as a therapeutic agent against SARS-CoV-2 have focused on several biological targets, including major protease (Mpro), spike protein (S protein), RdRp enzymes, human ACE2 receptor, nsp16 2′-O-methyltransferase, PLpro, HR1 and RBD, RNA-dependent RNA polymerase (RdRp), TMPRSS2, the receptor-binding domain (RBD) of SARS-CoV-2 and viral proteins such as helicase (Hel), exoribonuclease (ExoN) and guanine-N7 methyltransferase. These targets are central to the processes of viral replication, entry and infectivity, making them important areas of interest for potential antiviral interventions. Their efficacy of hesperidin’s mechanisms of action is evaluated on the basis of binding affinity, inhibitory effect on viral replication and potential antiviral activity. The results of all-publication analyses suggest promising interactions between hesperidin and critical residues of target proteins, indicating its potential as an inhibitor against SARS-CoV-2.

The S protein is of particular interest because it is the main means by which the virus enters host cells. By inhibiting them, hesperidin can prevent the virus from infecting cells. Similarly, inhibiting Mpro or RdRp may interfere with the virus’ ability to replicate, while modulating the hACE2 receptor may prevent the virus from binding to and entering host cells [43,44].

In silico studies have several advantages and limitations. The benefits of in silico research include its efficiency and speed, as it allows rapid evaluation of multiple compounds, against potential targets, which is much faster than conventional experimental methods, thus speeding up the initial stages of drug discovery. In addition, in silico studies are cost-effective because they reduce the need for costly laboratory experiments and clinical trials in the initial phases of research. They also offer safety benefits by eliminating the risks associated with early stage biological experiments, especially for hazardous materials or pathogens, and provide insight into the molecular interactions taking place, providing predictions of binding affinity, mechanisms of action and potential resistance mechanisms. Finally, in silico studies enable resource optimization through early identification of the most promising therapeutic candidates, which allows us to focus on experimental and clinical studies, optimizing both time and resources. In silico models offer valuable predictions, but these depend heavily on the accuracy of the algorithms and the quality of the data used. Incorrect assumptions or incomplete data can lead to misleading results that are not always consistent with actual biological behavior. The complexity of biological systems presents challenges for in silico research. Factors such as drug metabolism, immune responses and multi-target interactions can be difficult to simulate accurately, resulting in gaps between computational predictions and actual biological outcomes, and reliance on existing knowledge also limits the scope of in silico studies. To ensure the validity of in silico findings, it is necessary to verify them through laboratory experiments and clinical trials, which can be a time- and resource-intensive process. Additionally, in silico research requires sophisticated software and significant computing power [45,46].

Table 2 summarizes publications from 2020 to 2024, which investigated in silico hesperidin as a potential compound against SARS-CoV-2 [15,47,48,49,50,51,52,53,54,55,56,57,58,59,60,61,62,63,64,65,66,67,68,69,70,71,72,73,74,75,76,77,78]. Computational techniques used in the study include molecular docking, quantum chemical density functional theory (DFT) calculations, virtual screening, molecular dynamics simulations, quantitative structure-activity relationship analysis, structure-based virtual screening, molecular modeling techniques, predictive ADME studies, machine learning approaches, pharmacophore modeling and density functional theory. These methods are being used to analyze interactions, predict binding affinity, understand mechanisms of action and evaluate the efficacy of hesperidin in inhibiting SARS-CoV-2.

Efficacy results include binding affinity and stability in forming complexes with viral enzymes, potential multi-target inhibitory activity against SARS-CoV-2, non-competitive modulation and destabilization of interactions between viral spike protein and hACE2 receptor, interactions with key viral protein residues suggesting inhibitory potential, disruption of virus–host interaction and inhibition of viral replication, modulation of host immune response against SARS-CoV-2, inhibition of viral entry through interaction with key viral protein residues, potential as a multi-target inhibitor against SARS-CoV-2 and inhibition of SARS-CoV-2-hACE2 interaction, suggesting a possible role for hesperidin in preventing viral entry into cells. Hesperidin inhibits the SARS-CoV-2 virus spike protein through specific molecular interactions. By forming hydrogen bonds, hydrophobic interactions, electrostatic interactions and pi-stacking interactions with critical residues of the spike protein, hesperidin disrupts key protein-protein interactions necessary for virus attachment and entry. The mechanism includes recognition of specific residues, occupation of binding sites and overall inhibition of viral fusion and replication. The ability of hesperidin to target and interact with key residues of the spike protein hinders the infectivity of the virus by preventing it from effectively binding to the human ACE2 receptor [49].

The results of the analyses presented, suggest promising interactions between hesperidin and critical residues of target proteins, indicating its potential as an inhibitor against SARS-CoV-2.

## 6. Hesperidin Intake Levels and Some Potential Health Benefits

The daily intake of hesperidin is not currently subject to any specific recommendations, and the quantity consumed can vary greatly depending on individual dietary patterns and the consumption of hesperidin-rich foods. In research studies examining the health benefits of hesperidin, the administered amount can vary considerably based on factors such as the research objectives, health outcomes being investigated and the duration of the study. Perche et al. [79] undertook a research study to investigate the effects of orange juice consumption and hesperidin supplementation on the function of immune cells in healthy individuals. The study was conducted as a randomized crossover trial that lasted for a total of 18 weeks, divided into three treatment periods, each lasting for four weeks, with three-week washout periods in between. During each treatment period, the participants consumed either orange juice containing 292 mg of hesperidin, an isocaloric control drink with pure hesperidin or a placebo control drink. The study design involved monitoring the immune status of the participants at the start and end of each treatment period. The researchers used flow cytometry to assess immune cell function by examining leukocyte subpopulations based on cell surface markers, measuring cytokine secretion and evaluating reactive oxygen species (ROS) burst. Compliance with the study products was ensured by monitoring daily consumption and counting unconsumed products. Blood samples were collected at specific time points during the study to analyze immune parameters. The results indicated that orange juice consumption and hesperidin supplementation improved antioxidant capacity, immune status and DNA damage in the participants. The objective of the study conducted by Lorzdeh et al. [31] was to evaluate the impact of hesperidin supplementation on inflammatory markers using a systematic review and meta-analysis of randomized controlled clinical trials (RCTs). A random-effects model was used to compare the mean changes in inflammatory markers between the hesperidin-supplemented groups and the control subjects. This study included six eligible RCTs with a total of 296 participants. The meta-analysis results indicated that hesperidin significantly reduced vascular cell adhesion molecule 1 (VCAM-1) levels (weighted mean difference (WMD) = −22.81 ng/L, *p* = 0.041, n = 3). However, no significant changes were observed for serum C-reactive protein (CRP) levels (WMD = −0.69 mg/L, *p* = 0.079, n = 5); though, subgroup analysis revealed a significant reduction in studies with a parallel design (WMD = −0.72 mg/L, *p* = 0.024, n = 3) and those with more than four weeks of follow-up (WMD = −0.76 mg/L, *p* = 0.020, n = 2). Hesperidin supplementation had no significant effect on circulating E-selectin, interleukin 6 and Intercellular Adhesion Molecule 1 (ICAM-1) levels. The study concluded that while hesperidin supplementation significantly improved VCAM-1 levels, other inflammatory markers remained unaffected. Most studies tend to use 500 mg or more of supplemental hesperidin and use the standard form of hesperidin if taking it as a daily preventative. Yari et al. [80] conducted a randomized, double-blind, placebo-controlled clinical trial to evaluate the efficacy of hesperidin supplementation in normalizing metabolic abnormalities in individuals with metabolic syndrome (MetS). Participants with MetS were divided into two groups: one receiving 500 mg hesperidin twice daily, and the other receiving a placebo for 12 weeks. The results demonstrated that hesperidin supplementation resulted in significant reductions in fasting glucose levels, triglycerides, systolic blood pressure, TNF-α and hs-CRP levels as compared to the placebo group. Within-group analysis revealed significant decreases in the serum levels of glucose, insulin, triglycerides, total cholesterol, LDL cholesterol, TNF-α and hs-CRP in the hesperidin group, while only glucose and insulin significantly decreased in the control group. The study concluded that hesperidin supplementation could improve metabolic abnormalities and inflammatory status in individuals with MetS, thereby highlighting its potential as an adjuvant therapy in managing MetS and associated complications. Valls et al. [81] examined the influence of hesperidin on blood pressure regulation in individuals with pre- and stage-1 hypertension. The study recruited 159 participants from the general population, focusing on individuals aged 18–65 years with systolic blood pressure (SBP) ≥ 120 mmHg and no family history of cardiovascular disease or chronic illness. Participants were instructed to consume beverages with varying hesperidin contents over a 12-week period, with assessments conducted at regular intervals. The results indicated a dose-dependent decrease in SBP and pulse pressure (PP) with the hesperidin content of the beverage consumed. Notably, a single dose of 500 mL of hesperidin-enriched orange juice led to significant reductions in SBP and PP, suggesting acute blood pressure-lowering effects. The study also highlighted the relationship between changes in gene expression, such as PTX3 and NAMPT, and blood pressure outcomes. The study’s open access nature under a Creative Commons Attribution 4.0 International License allows for widespread sharing and adaptation of the research findings, contributing to the body of knowledge on the health-promoting effects of citrus flavanones like hesperidin. Tadros et al. [82] studied the effects of hesperidin in 100% orange juice on chronic disease biomarkers such as inflammation, neurological function, cardiac health, insulin levels, antioxidant/phenolic levels and oxidative stress biomarkers. The daily recommended intake of hesperidin was reported to range from 0.0025 to 582.5 mg per day.

## 7. Hesperidin Contents in Some Citrus Juices

Citrus fruits, such as oranges, grapefruits, lemons, limes, and mandarins, all possess hesperidin in various amounts. Oranges, including both sweet and bitter varieties, are renowned for their significant hesperidin content, which can range from 30 to 177 mg/100 mL depending on the specific variety and cultivation conditions. Grapefruits, while containing hesperidin, have lower levels compared to oranges, with juice hesperidin content ranging from 3 to 7 mg/100 mL. Lemons and lemon juice have lower hesperidin content than oranges and grapefruits, typically ranging from 0.5 to 4 mg/100 mL. Mandarins, including tangerines, contain moderate amounts of hesperidin, with mandarin juice content varying widely and falling within the range of 20 to 80 mg/100 mL, depending on the variety and maturity at harvest. Limes have a relatively low content of hesperidin, the amount of which usually ranges from 0.5 to 3 mg/100 mL. These values highlight the variability in hesperidin content across citrus fruits. Factors such as specific citrus varieties, cultivation methods, ripeness at the time of juice extraction, and processing techniques can influence the final hesperidin concentration in the juice. Generally, hesperidin content is higher in the peel and membranous parts of these fruits, and juices with higher pulp content tend to contain more hesperidin [17,83,84]. To enhance the delivery of hesperidin from dietary sources, several strategies can be considered, taking into account factors that affect its bioavailability. Some food processing techniques, such as juicing or blending citrus fruits, can disrupt plant cell walls and release bound hesperidin, thereby increasing its bioavailability. Concomitant administration of hesperidin with compounds that enhance its absorption, such as vitamin C, other flavonoids, may improve bioavailability by affecting efflux and transport mechanisms. Modulating the intestinal microbiota with prebiotics, probiotics, or synbiotics can affect hesperidin metabolism and increase its bioavailability. Combining hesperidin with absorption enhancers such as piperine or quercetin may improve its absorption by inhibiting efflux transporters and increasing intestinal permeability [35,85].

## 8. Citrus Fruits Consumption in China

China is known for producing a substantial quantity of total citrus fruits, which surpasses that of many other nations. In 2019, the production volume of citrus fruits, including oranges, lemons, tangerines and grapefruits, reached considerable levels, with the total amount surpassing approximately 46 thousand tons and in 2022–2060. China’s considerable domestic market for citrus fruits, due to its large population, results in high levels of consumption within this country. Guangxi province currently represents about 30 percent of China’s citrus production, while Hunan and Hubei account for 20 percent. Reports suggest a notable upsurge in the consumption of citrus fruits and their juices, particularly orange juice, in China. This trend is largely ascribed to the growing public awareness of the health benefits of vitamin C and their desire to boost their daily intake of vitamin C. Despite the widely held belief in the protective effects of vitamin C, epidemiological data from China during the COVID-19 pandemic indicate a substantial incidence of SARS-CoV-2 infection, suggesting that increased consumption of citrus fruits alone may not significantly affect the virus [86,87,88,89,90].

## 9. Limitations and Challenges in Interpreting Findings from Clinical, In Vitro and In Silico Studies

The primary impediment to utilizing hesperidin as a compound against SARS-CoV-2 is the disparity between the concentrations of hesperidin utilized in laboratory experiments in vitro and clinical studies (typically three times daily 500 mg per os) and the amounts that can be obtained from consuming citrus fruits. In order to achieve such concentrations, an individual would have to drink approximately half a liter of juice, such as orange juice, at a time. This raises significant questions about the direct application of the results of these studies to the delivery of hesperidin to the body from citrus fruits. Another significant challenge is the low bioavailability of hesperidin, which is highly variable and dependent on factors such as individual metabolism and gut microflora. This variability makes it difficult to determine an effective dose for therapeutic benefit and challenging to extrapolate laboratory findings to broader human populations. A comparison of epidemiological studies with citrus fruit consumption data in China between 2020 and 2024 did not reveal a causal relationship between hesperidin provided from these fruits and a reduced incidence of COVID-19. The potential protective effect could be due to the synergistic action of multiple bioactive compounds present in citrus fruits, rather than hesperidin alone. The complexity of dietary patterns and the presence of various confounding factors further complicate the interpretation of these results. Factors such as cultural practices, socioeconomic status and individual health conditions strongly influence dietary habits, making it difficult to isolate the specific effect of hesperidin on COVID-19 cases. In silico studies, on the other hand, provide valuable insights into potential hesperidin interactions with SARS-CoV-2, but these are not always consistent with actual biological results due to the complex behavior of viruses in the host and the complexity of human biology.

## 10. Future Directions

Additional clinical trials are necessary to fully evaluate the efficacy and safety of hesperidin in the context of COVID-19. These studies should focus on determining the optimal dose, enhancing bioavailability and evaluating the therapeutic effects of hesperidin in various human populations. Although the in vitro studies presented in this article have demonstrated promising antiviral properties of hesperidin, it is crucial to conduct comprehensive testing to translate these effects to clinical settings. It is also important to monitor potential side effects and interactions with other drugs to ensure the safe use of hesperidin. To maximize the therapeutic efficacy of hesperidin, it is essential to understand and improve its bioavailability. Future research should explore new formulation strategies for hesperidin that could increase its bioavailability, such as the use of complexing agents or encapsulation techniques. Expanded epidemiological studies are necessary to establish a clear link between hesperidin consumption and COVID-19 outcomes. These studies should include larger and more diverse populations to provide a broader understanding of the role of hesperidin under these conditions. Efforts should be made to control for confounding factors and to account for the complexity of dietary patterns and lifestyle habits.

It is essential to conduct more research into the molecular mechanisms by which hesperidin exerts its antiviral effects besides in silico studies. Understanding the interactions between hesperidin and SARS-CoV-2, as well as its impact on the immune system, is crucial for developing targeted therapeutic strategies. Future studies should explore the potential synergistic effects of hesperidin and other bioactive compounds, as also suggested by Gupta et al. [73]. Longitudinal studies that track dietary hesperidin intake and its health effects over time may provide valuable insights into its long-term effects and role in preventing or alleviating viral infections, including COVID-19. Such studies are essential for assessing the lasting impact of regular hesperidin consumption on health and immunity. Collaborative and multidisciplinary research efforts involving experts in virology, immunology, nutrition and pharmacology are necessary to address the challenges posed by SARS-CoV-2 and to fully exploit the potential of hesperidin as an agent against this virus.

## 11. Conclusions

The study of hesperidin as a potential agent against SARS-CoV-2 has opened new avenues for the search for effective strategies to combat the COVID-19 pandemic. In vitro, in silico and clinical studies conducted between 2020 and 2024 have provided valuable information on the antiviral properties of hesperidin, including its ability to inhibit viral entry, interfere with viral replication and modulate immune response. The discrepancy between laboratory and dietary concentrations of hesperidin, the complexity of its bioavailability and metabolism and the need to control confounding factors in epidemiological studies are significant hurdles that must be addressed. Future research, including more clinical trials and comprehensive epidemiological studies, is needed to overcome these challenges and to establish the efficacy and safety of hesperidin in SARS-CoV-2 infections. The potential implications of hesperidin in public health and therapeutic strategies are far-reaching. Dietary recommendations that emphasize the consumption of citrus fruits rich in hesperidin, development of hesperidin-based supplements and inclusion of hesperidin in therapeutic protocols may modify approaches to viral infections, including COVID-19. Public health policies and education focused on the benefits of bioactive compounds such as hesperidin may also play a key role in improving overall health and preventing viral disease. Hesperidin is emerging as a promising candidate in the fight against COVID-19, with the potential to contribute to both preventive and therapeutic strategies. Although further research is needed to fully understand and exploit its potential, the current findings provide a foundation for future research and development.

## Figures and Tables

**Table 1:** General summary of clinical trials on hesperidin as potential compound against SARS-CoV-2.

Reference	Trial ID	Year	Study Design	Study Population	Duration	Control	Outcome Measures	Results
[36]	NCT04715932	2022	Randomized, double-blind, placebo-controlled	216	14 days	Placebo	Yes	Yes
[37]	NCT04452799	2020	Randomized double-blind	100	14 days	No treatment	Yes	Yes
[38]	IRCT20150725023332N5	2022	Randomized controlled	20	5 days	No treatment	Yes	Yes

**Table 2:** General summary of research topics and results on hesperidin as potential compound against SARS-CoV-2 in silico studies.

References	Computational Techniques	Biological Targets of SARS-CoV-2	Effectiveness Outcomes
[15]	1. Molecular docking 2. Molecular dynamic simulations	Mpro, S protein, RdRp, nsp13	1. Strong binding affinity with viral proteins
[48]	1. Molecular docking 2. Quantum chemical density functional theory calculations	Mpro, S protein	1. Binding affinity 2. Inhibitory effects on viral replication 3. Comparative analyses with standard antiviral drugs
[49]	1. Molecular docking	S protein, hACE2	1. Interactions with key residues of the spike protein and ACE2 receptor
[50]	1. Molecular docking 2. Molecular dynamics simulations	S protein, hACE2	1. Non-competitive modulator that destabilizes the interaction between the spike protein and the ACE2 receptor
[51]	1. Molecular docking 2. Virtual screening	Mpro, hACE2, PLpro, HR1, RBD	1. Affinity to bind 2. Interact with key viral proteins 3. Interfere with virus-host interactions, inhibit viral replication
[52]	1. Molecular docking 2. Molecular dynamics simulations	Mpro, S protein, RdRp, TMPRSS2, hACE2	1. Inhibitory effect on virus replication, entry and infectivity 2. Potential to modulate host immune response against SARS-CoV-2
[53]	1. Molecular docking 2. Molecular dynamics simulations	S protein, RdRp	1. Binding affinity, stability and potential inhibitory effect on viral proteins
[54]	1. Gaussian09 software for electronic calculations 2. Density functional theory 3. Conceptual density functional theory for antioxidant properties	Mpro, S protein, RdRp	1. Evaluation of the ability to interact with viral components 2. Potential inhibition of viral replication
[55]	1. Molecular docking 2. Molecular dynamics simulations	Mpro	1. Binding affinity to viral proteins, particularly Mpro 2. Potential inhibitor of viral replication and maturation
[56]	1. Molecular docking 2. Molecular dynamics simulations	Mpro	1. Binding energy, binding sites, key interactions with viral proteins
[57]	1. Molecular docking 2. Molecular dynamics simulations 3. Pharmacokinetic studies	S protein	1. Inhibiting viral proteins or disrupting viral-host interactions, as evidenced by favorable binding affinities, pharmacokinetic properties 2. Potential inhibitory effects on viral entry or replication.
[58]	1. Molecular docking 2. Molecular dynamics simulations 3. MM-GBSA analysis	Mpro, S protein, RdRp, N protein, E protein	1. Binding energy values and key residue interactions 2. Drug-likeness assessments 3. ADMET properties
[59]	1. Molecular docking 2. Molecular dynamics simulations 3. Virtual screening 4. Quantitative structure-activity relationship analysis	S protein, RBD, hACE2	1. Key interactions identification 2. Binding energies 3. Inhibition constants and mechanism of action
[60]	1. Molecular docking 2. Structure-based virtual screening	RBD, hACE2	1. Inhibit the SARS-CoV-2-ACE2 interaction, suggesting a possible role in preventing viral cellular entry
[61]	1. Molecular docking 2. Molecular dynamics simulations 3. Free energy calculations 4. Target prediction algorithms	Mpro, RdRp	1. Binding affinity 2. Stability of protein-ligand complexes 3. Inhibitory activity against viral proteins
[62]	1. Molecular docking 2. Blind docking analyses	Mpro	1. Estimated free energy of binding for the main protease
[63]	1. Molecular docking 2. Molecular dynamics simulations 3. Virtual screening 4. Deep learning tools for drug-target interaction predictions	Mpro	1. Potential inhibitor of SARS-CoV-2. Targeting key viral proteins
[64]	1. Molecular docking 2. Molecular dynamics simulations	S protein	1. Binding affinity, stability, and specific interactions with viral
[65]	1. Molecular docking 2. Molecular dynamics simulations 3. Binding free energy calculations	Mpro, S protein	1. Inhibiting viral replication 2. Blocking viral entry into host cells 3. Modulating the host immune response
[66]	1. Molecular docking 2. Machine learning approaches	Mpro, S protein	1. Potential inhibitor of Mpro 2. Binding interactions and potential antiviral activity
[67]	1. Molecular docking 2. Molecular dynamics simulations	Mpro, S protein, hACE2	1. Binding affinity scores 2. Interaction energies 3. Key residues involved in hesperidin-protein interactions 4. Constant inhibition or IC50 values for quantifying the potency of hesperidin as an antiviral agent
[68]	1. Molecular docking 2. Molecular dynamics simulations	Mpro	1. Promising binding energies 2. Interactions at Mpro active site
[69]	1. Molecular docking 2. Binding affinity tests, including biolayer interferometry assay and isothermal titration calorimetry assay	Mpro, hACE2, S protein, RBD	1. Binding affinity with ACE2, M, S, RBD proteins 2. Impact on immune, inflammation, virus infection, IC50 values (51.5 μM and 5.5 mM)
[70]	1. Molecular docking 2. Molecular dynamics simulations	Mpro, S protein, hACE2	1. Binding energies 2. Interaction patterns 3. Key amino acid residues 4. Evaluate stability and dynamics of complexes
[71]	1. Molecular docking 2. Molecular dynamics simulations 3. Pharmacophore modeling	Mpro, S protein, RdRp, PLpro, nsp13	1. Evaluate binding affinity, stability, potential to inhibit viral replication
[72]	1. Molecular docking 2. Molecular dynamics simulations 3. SwissADME and ProTox-II for drug-likeness and toxicity assessment	Mpro, TMPRSS2, PLpro	1. Strong complex formation 2. Stable interactions with viral proteins
[73]	1. Molecular docking 2. Molecular dynamics simulations 3. Molecular modeling techniques	nsp13, ExoN, Guanine-N7 methyltransferase	1. Interactions with critical residues of target proteins
[74]	1. Molecular docking 2. Molecular dynamics simulations	Mpro, RdRp	1. Binding affinity 2. Stability in forming complexes with viral enzymes 3. Potential multi-target inhibitory activity
[75]	1. Molecular docking 2. Molecular dynamics simulations 3. Virtual screening	nsp16,2′-O-methyltransferase	1. Promising interactions with key residues of the nsp16 protein
[76]	1. Molecular docking 2. Molecular dynamics simulations 3. ADMET for drug properties	Mpro, RdRp	1. Superior binding affinities with Mpro, RdRp compared to standard drugs 2. Strong interactions with catalytic residues
[77]	1. Molecular docking 2. Molecular dynamics simulations	Mpro, S protein, hACE2	1. Inhibitory effects on viral proteins 2. Disruption of viral entry mechanisms 3. High binding affinities to key viral targets
[78]	1. Molecular docking 2. Molecular dynamics simulations	Mpro, allosteric site	1. Identified potent allosteric inhibitors

Mpro—SARS-CoV-2 main proteinase; S protein—SARS-CoV-2 spike glycoprotein; hACE2—human angiotensin-converting enzyme 2 receptor; RdRp—RNA-dependent RNA polymerase; nsp13—non-structural protein 13 (helicase); PLpro—papain-like protease; HR1—heptad repeat 1 domain; RBD—receptor binding domain; TMPRSS2—transmembrane serine protease 2; N Protein—ucleocapsid protein; E protein—envelope protein; ExoN—exoribonuclease.
